# Optogenetic Control of Neural Circuits in the Mongolian Gerbil

**DOI:** 10.3389/fncel.2018.00111

**Published:** 2018-04-24

**Authors:** Stefan Keplinger, Barbara Beiderbeck, Stylianos Michalakis, Martin Biel, Benedikt Grothe, Lars Kunz

**Affiliations:** ^1^Division of Neurobiology, Department Biology II, Biocenter, Ludwig Maximilian University of Munich, Munich, Germany; ^2^Graduate School of Systemic Neurosciences, GSN-LMU, Ludwig Maximilian University of Munich, Munich, Germany; ^3^Center for Integrated Protein Science Munich (CiPSM), Department of Pharmacy, Center for Drug Research, Ludwig Maximilian University of Munich, Munich, Germany

**Keywords:** auditory, brainstem, AAV, MNTB, DNLL, IC, CatCH, NpHR3.0

## Abstract

The Mongolian gerbil (*Meriones unguiculatus*) is widely used as a model organism for the human auditory system. Its hearing range is very similar to ours and it uses the same mechanisms for sound localization. The auditory circuits underlying these functions have been characterized. However, important mechanistic details are still under debate. To elucidate these issues, precise and reversible optogenetic manipulation of neuronal activity in this complex circuitry is required. However, genetic and genomic resources for the Mongolian gerbil are poorly developed. Here, we demonstrate a reliable gene delivery system using an AAV8(Y337F)-pseudotyped recombinant adeno-associated virus (AAV) 2-based vector in which the pan-neural human synapsin (hSyn) promoter drives neuron-specific expression of CatCH (Ca^2+^-permeable channelrhodopsin) or NpHR3.0 (*Natronomonas pharaonis* halorhodopsin). After stereotactic injection into the gerbil’s auditory brainstem (medial nucleus of the trapezoid body, dorsal nucleus of the lateral lemniscus) and midbrain [inferior colliculus (IC)], we characterized CatCH- and/or NpHR3.0-transduced neurons in acute brain slices by means of whole-cell patch-clamp recordings. As the response properties of optogenetic tools strongly depend on neuronal biophysics, this parameterization is crucial for their *in vivo* application. In a proof-of-principle experiment in anesthetized gerbils, we observed strong suppression of sound-evoked neural responses in the dorsal nucleus of the lateral lemniscus (DNLL) and IC upon light activation of NpHR3.0. The successful validation of gene delivery and optogenetic tools in the Mongolian gerbil paves the way for future studies of the auditory circuits in this model system.

## Introduction

The discovery of channelrhodopsins and light-activated Cl^-^ pumps and their application as highly versatile optogenetic tools for controlling light-stimulated excitation and inhibition of neurons have made many exciting neurobiological discoveries possible over the past decade (Boyden et al., 2005; Nagel et al., 2005; Deisseroth et al., 2006; Zhang et al., 2010; Fenno et al., 2011; Yizhar et al., 2011a). Since the application of optogenetics requires gene transfer, most *in vivo* studies of neural networks have been performed in the mouse, for which methods of gene transfer and genome manipulation are well established. For model organisms such as the Mongolian gerbil (*Meriones unguiculatus*), the use of optogenetic tools is far less well developed. The Mongolian gerbil is the model of choice for human hearing research because – unlike the case in mice and rats – its audiogram includes most of the human low-frequency hearing range (Ryan, 1976) and age-related hearing loss occurs in both species (Mills et al., 1990). The circuitry underlying sound source localization has been well described *in vitro* and *in vivo* and resembles that found in humans (Spitzer and Semple, 1991, 1995; Tollin, 2003; Maki and Furukawa, 2005; Siveke et al., 2006; Pecka et al., 2008; Kandler et al., 2009; Lingner et al., 2012; Grothe and Pecka, 2014; Roberts et al., 2014; Manley, 2017). Unfortunately, annotation of the gerbil genome is only now on its way (Zorio et al., 2018). and techniques for the generation of transgenic or knock-out strains are difficult because breeding is time consuming and reproductive performance is poor compared to mice (Ågren, 1984; Salo and French, 1989).

The *in vivo* application of optogenetic tools to the Mongolian gerbil would enable us to understand functions of auditory circuits by reversibly activating or silencing defined brain nuclei (Willaredt et al., 2015; Nothwang, 2016). In the context of sound source localization, the ability to optogenetically address the MNTB, the dorsal nucleus of lateral lemniscus (DNLL) and the IC would be a huge step forward, as these nuclei are crucial for auditory functions. The MNTB is central to sound source localization because it provides glycinergic inhibition to the MSO and the LSO. Moreover, it represents a good example for a nucleus in which direct electrical stimulation of neurons is impossible, because large numbers of fibers projecting to the MSO and LSO pass through it and would be electrically activated as well. The DNLL has been hypothesized to be crucial in the context of echo suppression and the precedence effect (Cremer, 1948; Wallach et al., 1949; Haas, 1951; Shneiderman et al., 1988; Ito et al., 1996; Kelly et al., 1996; Pecka et al., 2007; Ammer et al., 2015). The IC is a central structure in sound processing (Pollak et al., 2003). It represents an ideal model nucleus because it is easily accessible to both virus injection and light application, due to its dorsal location.

In order to express channelrhodopsins and halorhodopsins in designated auditory nuclei, we chose recombinant AAV vectors as a reliable gene delivery system. AAV vectors are easy to produce in high titers and safe to use owing to their lack of pathogenicity, low immunogenicity, replication deficiency and low biological safety level. More than 12 naturally occurring subtypes have been described, which display distinct tropisms for certain cell types (Burger et al., 2004; Zincarelli et al., 2008; Aschauer et al., 2013). Among the various serotypes, AAV8 and AAV9 transduce neurons at very high rates (Broekman et al., 2006; Masamizu et al., 2011). Moreover, phosphorylation of surface-exposed tyrosine residues in the AAV capsid has been reported to contribute to targeting to the proteasome for degradation (Zhong et al., 2008). Accordingly, AAV vectors with mutated tyrosine residues may enhance levels of gene expression. To restrict gene expression to neuronal cells, we used the 485-bp hSyn promoter. AAVs have previously been used to transduce regions in the frontal cortex and the hippocampus in the gerbil (Shimazaki et al., 2000; Bellomo et al., 2006). In a recent study, an AAV9 vector bearing an NpHR gene (see below) was utilized to silence firing of the trigeminal nerve in the gerbil brainstem (Chen et al., 2017). However, nuclei in the midbrain or brainstem of gerbils have remained inaccessible to optogenetic manipulations. We chose AAV8(Y337F), an AAV8 serotype with a single tyrosine-to-phenylalanine mutation at position 733 in the AAV viral protein 1 (VP1), as the vehicle for gene transfer. The vector of choice must provide for: (1) specific and efficient transduction of neurons in the auditory midbrain and brainstem, (2) reliable expression of a functional transgene in neurons, (3) fast (≤2 weeks), stable and high-level gene expression to permit manipulation of the membrane potential upon expression of optogenetic tools, and (4) transduction of sufficiently high numbers of neurons (i.e., at least 30–50%) within the injected area.

For neuronal excitation, we utilized the L132C mutant ChR2 variant CatCH (Ca^2+^ translocating channelrhodopsin) (Kleinlogel et al., 2011). CatCH exhibits a three–fourfold increase in photocurrent relative to wild-type ChR2, due to a shift in the balance of the pore’s ion selectivity filter in favor of Ca^2+^, a small shift in absorption maximum (474 nm), a >10-fold increase in light sensitivity, and fast kinetics (τ_off_ = 16 ms). For inhibition, we used the light-driven chloride pump NpHR3.0, which is based on the NpHR, the halorhodopsin isolated from the archeal halobacterium *Natronomonas pharaonis* (Han and Boyden, 2007; Zhang et al., 2007). It has an absorption maximum at 590 nm and hyperpolarizes the cell membrane by pumping chloride into the cytoplasm. Targeting of NpHR3.0 to the plasma membrane was improved and its aggregation prevented by adding the trafficking signal from K_ir_2.1, an inward rectifying K^+^ channel (Gradinaru et al., 2010). NpHR3.0 features an increased photocurrent of ∼750 pA, fast kinetics with a τ_off_ of ∼4.2 ms and a 20-fold increase in light sensitivity compared to the wild-type NpHR. Following successful gene delivery and expression, CatCH and NpHR3.0 needed to be characterized *in vitro* to determine the appropriate expression times, success rate, optimal light stimulation protocols, the kinetics of antagonist action and their effects on neuronal spiking. These issues were addressed by obtaining whole-cell patch-clamp recordings from transduced neurons in acute brain slices. In a final proof-of-principle experiment involving extracellular *in vivo* recordings, sound-evoked neuronal spiking in the IC and the DNLL of anesthetized animals was suppressed by light in neurons expressing NpHR3.0.

## Materials and Methods

### Gerbils

Ethical approval was obtained for the animal work. The experiments were carried out in accordance with regional regulations, national law (German Animal Welfare Act) and the Council of the European Union’s Directive 2010/63/EU. All experiments were approved by the District Government of Upper Bavaria (‘Regierung von Oberbayern’; Ref. No. 55.2-1-54-2531-105-10). Mongolian gerbils (*M. unguiculatus*) were bred in the certified in-house breeding facility at LMU Munich (German Animal Welfare Act, 4.3.2-5682/LMU/Department Biology II). Gerbils of either sex were injected with AAVs on postnatal day (P) 28-60 for *in vivo* recordings and immunohistological analyses and on P 2-30 for *in vitro* experiments (Keplinger, 2016).

### Construction of hSyn-CatCH-mCherry-WPRE

pAAV2-ss-hSyn-hChR(H134R)-mCherry-WPRE (obtained from Dr. Karl Deisseroth) served as backbone for vector construction and was cleaved with SalI and HindIII (New England Biolabs Inc.). The DNA coding for CatCH (from Dr. Sonja Kleinlogel) was PCR-amplified with the primers 5′-TATAGTCGACATGGATTATGGAGGCGC-3′ and 5′-GGCTGGCGCGGTACCCAAGCTTAT-3′. The PCR product was cleaved with SalI and HindIII, and ligated into the corresponding sites in the dephosphorylated vector plasmid. The ligation mixture was transformed into *Escherichia coli* 10-beta cells and positive transformants were selected by restriction fragment length analysis. The intact plasmid pAAV2-ss-hSyn-CatCH-WPRE was then cleaved with HindIII and dephosphorylated. mCherry was PCR-amplified with the primers 5′-TATAAAGCTTATGGTGAGCAAGGGCG-3′ and 5′-CGAGCTGTACAAGTAAAAGCTTAT-3′. The PCR product was digested with HindIII and ligated into the same site in pAAV2-ss-hSyn-CatCH-[/]-WPRE. The ligation was transformed into the *E. coli* 10-beta, and positive clones with the correct plasmid were identified by digesting isolated DNA.

### Construction of pAAV2.1-ss-hSyn-Catch-mCherry-T2A-NpHR3.0-WPRE

The donor plasmid described above was digested with SalI and EcoRI, and the new insert CatCH-mCherry-T2A-NpHR was constructed by fusion PCR. The three parts of the insert, CatCH-mCherry, T2A and NpHR3.0 were amplified by PCR. CatCH-mCherry was amplified with the primers 5′-TATAGTCGACATGGATTATGGAGGCGC-3′ and 5′-CTTGTACAGCTCGTCCATGCC-3′. T2A DNA was amplified with the primers 5′-GCGGCATGGACGAGCTGTACAAGGCCACGAACTTCTCTCTGTTAAA-3′ and 5′-AGGACCGGGGTTTTCTTCCA-3′. NpHR3.0 was amplified with the primers 5′-GACGTGGAAGAAAACCCCGGTCCTATGACAGAGACCCTGCCTCCC-3′ and 5′- CTCTGAATTCTTTACACCTCGTTCTCGTAGCAGAACACGTTGATGTCGATCTGGTCC-3′. All three amplicons were purified by agarose gel electrophoresis. T2A and NpHR3.0 were fused together using the primers 5′-GCGGCATGGACGAGCTGTACAAG GCCACGAACTTCTCTCTGTTAAA-3′ and 5′-CTCTGAATTCTTTACACCTCGTTCTCGTAGCAGAACACGTTGATGTCGATCTGGTCC-3′. CatCH-mCherry and T2A-NpHR3.0 were fused with the primers 5′-TAGAGTCGACACTATGGCGGCGCTTTGTCTG and 5′-CTCTGAATTCTTTACACCTCGTTCTCGTAGCAGAACACGTTGATGTCGATCTGGTCC-3′. The resulting amplicon CatCH-mCherry-T2A-NpHR3.0 was then purified by agarose gel electrophoresis and subcloned into the cloning vector pJet1.2. The ligation was transformed into *E. coli* 10-beta. The insert was then cut out of its host vector with SalI and EcoRI, and ligated into the cleaved donor plasmid.

### AAV Vector Production

The *cis* plasmids pAAV2.1-ss-hSyn-EYFP-WPRE, pAAV2.1-ss-hSyn-CatCH-mCherry-WPRE, pAAV2.1-ss-hSyn-NpHR3.0-EYFP-WPRE, pAAV2.1-ss-hSyn-Catch-mCherry-T2A-NpHR3.0-WPRE, and pAAV2.1-sc-hSyn-NpHR3.0-mCherry-SV40 were used to produce single-stranded AAV8(Y733F)-pseudotyped AAV2 vectors according to published procedures (Koch et al., 2012; Becirovic et al., 2016). Briefly, 293T cells were transfected with the *trans* plasmids pAdDeltaF6, and pAAV2/8Y733F, and the appropriate *cis* plasmid, using the calcium phosphate method. rAAV2/8Y7333F particles were harvested after 48 h and purified on iodixanol gradients. The 40–60% iodixanol interface was further purified and concentrated by ion-exchange chromatography on a 5-ml HiTrap Q Sepharose column using the ÄKTA Basic FPLC system (GE Healthcare, Munich, Germany) according to previously described procedures, followed by further concentration using Amicon Ultra-4 Centrifugal Filter Units (Millipore, Schwalbach, Germany). Physical titers (in genome copies/ml) were determined by quantitative PCR on a LightCycler 480 (Roche Applied Science, Mannheim, Germany) using the KAPPA SYBR FAST kit (Peqlab, Erlangen, Germany) and the following primer set: WPREF: 5′-AGTTGTGGCCCGTTGTCAGG-3′ and WPRER: 5′-AGTTCCGCCGTGGCAATAGG-3′. In all our experiments, the vector with the DNA backbone of the AAV2 serotype was packaged in the capsid of the rAAV2/8Y733F, and in the following this combination is referred to as AAV8(Y733F).

### AAV Injection

Gerbils were anesthetized by subcutaneous injection with medetomidine (Domitor, Pfizer Inc.), midazolam (Midazolam, Ratiopharm GmbH) and fentanyl (Fentanyl, Janssen Pharmaceutica), and additionally injected with meloxicam (Metacam) and Ringer’s solution (B. Braun Melsungen AG). Anesthetized animals were placed on a custom-made heating pad and the depth of anesthesia was monitored by testing the paw pinch reflex. An incision was made from lambda to 1 cm rostral of the bregma and a custom-made head-fixating device was glued onto the skull directly rostral to the bregma. Gerbils were then head fixed in a custom-made stereotaxic frame (Schuller et al., 1986), which allowed motorized movement in X-, Y- and Z-direction by micro manipulators (Junior XYZ-R, Luigs & Neumann Feinmechanik und Elektrotechnik GmbH) controlled by a XYZ axis controller (SM-5, Luigs & Neumann Feinmechanik und Elektrotechnik GmbH). The skull was aligned relative to lambda and bregma. A small 2-mm hole was drilled into the skull without damaging the dura mater. Injection needles with an inner diameter of 30–40 μm at the tip were pulled with a vertical glass electrode puller (PE-2, Narishige Ltd.) from glass capillaries (ID = 0.530 mm ± 25 μm, OD 1.14 mm, Item#: 4878, World Precision Instruments Inc.). They were filled with mineral oil, inserted into the micro injector (Nanoliter 2000, World Precision Instruments Inc.) and front-filled with 1 μl rAAV (diluted to 9.5–9.8 × 10^8^gc/μl). The position of the tip was then zeroed at lambda and lowered with 10 μm/sec to the respective coordinates (relative to lambda: IC *x* = 1900 μm, *y* = 0 μm, *z* = -3900/-4000 μm, injector angle: 20° to rostral; DNLL *x* = 1750 μm, *y* = -900 μm, *z* = -5400/-5500 μm, injector angle: 4–5° to medial; MNTB *x* = 1050 μm, *y* = 950 μm, *z* = -8050/-8150 μm, injector angle 12° to rostral). The injection (final volume 250 nl per injection spot) was delivered at a rate of 0.92 nl/sec. After 5 min, the needle was retracted (10 μm/sec), the gerbil was removed from the stereotaxic frame and the incision was sutured (Suprama HS18 USP 4/0, Feuerstein GmbH) and glued with histoacryl. Anesthesia was antagonized with atipamezole (Antisedan, Orion Pharma), flumazenil (Anexate, Roche Pharma AG) and naloxone (Naloxon, Hameln Pharma Plus GmbH), and Ringer’s solution was administered by subcutaneous injection. The gerbils were monitored closely until recovery and meloxicam was administered at 24-h intervals for at least 3 days after surgery. The success rates for injection into the target nuclei, i.e., the presence of transduced neurons afterwards, were 100% for the IC (*n* = 12/12), 33% for the DNLL (*n* = 1/3) and 33% for the MNTB (*n* = 1/3).

### Immunohistochemistry and Confocal Microscopy

Immunohistochemistry was carried out on tissue obtained from animals between P 28-60. The animals were injected with a lethal dose of pentobarbital [Narcoren (160 g/l), Merial GmbH; 2 mg/kg body mass] and perfused with PBS containing 0.1% heparin and 155 mM NaCl, followed by perfusion with 4% paraformaldehyde (PFA). After perfusion, the brain was removed from the skull and post-fixed in 4% PFA either overnight for vibratome sectioning or for 2–4 h for cryosectioning. In the case of cryosectioning, the brains were incubated in 20% sucrose solution (diluted in 0.02 M PBS) overnight to prevent solidification after freezing. They were then washed in 0.02 M PBS and deep-frozen using high-pressure CO_2_. Cryosections of 50-μm thickness were cut using a Leica CM3050 S cryostat. For vibratome sectioning brains were washed twice in 0.1 M PBS and slices of 50-μm thickness were cut with a VT1200S (Leica).

Standard immunohistochemistry procedures were used to stain free-floating slices with primary antibodies for NeuN (Millipore, MAB377), S100B (SWANT, 37A), and MAP2 (Neuromics, CH22103). Secondary antibodies were applied the following day for 2 h at room temperature. These were conjugated with Alexa 488 (Molecular Probes, Invitrogen), Cy3 (Dianova) or Cy5 (Dianova). Slices were mounted in Vectashield medium (H-1200, Vector Laboratories) and confocal scans were taken with a Leica SP5 System. Images were acquired with 8-bit intensity resolution using a HCX PL APO CS 20.0x0.70 IMM UV oil-immersion objective (resulting voxel size: 0.7568 μm × 0.7568 μm × 0.6294 μm) or with a HCX PL APO lambda blue 63.0 × 1.40 OIL UV oil-immersion objective (resulting voxel size: 0.2403 μm × 0.2403 μm × 0.9651μm).

### Image Data Analysis

Confocal stacks were analyzed with ImageJ^1^ and the following installed plug-ins: Biovoxxel extensions (Jan Brocher), Bio-Formats extension (LOCI), Cell Counter Plug-in (Kurt de Vos). Z-stacks of every 2nd horizontal brain section were made and the slices in each z-stack were summed into z-projections. To derive a mask encompassing the area of rAAV transduction a gaussian blur (*r* = 20 μm) filter was applied to the thresholded z-projection of the EYFP channel (threshold algorithm: mean; threshold value: 5x background noise intensity in the EYFP channel). This image was then converted into a binary mask and the mask saved as region of interest (ROI) in ImageJ. The EYFP sum z-projection was overlaid with the ROI now encompassing the transduction area. The transduction area (=area within ROI) was measured using the standard built-in tools in ImageJ. Total neurons (neuronal marker) and transduced neurons (EYFP) were manually counted inside the transduction area with the help of the Cell Counter plug-in.

### *In Vitro* Electrophysiology

Gerbils (10–28 days post injection) were anesthetized with Isofluran (IsoFlo, Abbot Laboratories) and decapitated. Brains were removed and kept in slice solution (50 mM sucrose, 25 mM NaCl, 25 mM NaHCO_3_, 2.5 mM KCl, 1.25 mM NaH_2_PO_4_, 3 mM MgCl_2_, 0.1 mM CaCl_2_, 25 mM glucose, 0.4 mM ascorbic acid, 3 mM *myo*-inositol and 2 mM sodium pyruvate; Sigma-Aldrich Chemie GmbH) bubbled with 95% O_2_ and 5% CO_2_. Horizontal brainstem sections of 200 μm thickness were cut using a VT1000S or VT1200S vibratome (Leica Biosystems) and incubated in extracellular recording solution (25 mM NaHCO_3_, 2.5 mM KCl, 1.25 mM NaH_2_PO_4_, 125 mM NaCl, 25 mM glucose, 0.4 mM ascorbic acid, 3 mM *myo*-inositol, 2 mM sodium pyruvate, 2 mM CaCl_2_, and 1 mM MgCl_2_; Sigma-Aldrich Chemie GmbH) at 36°C for 45 min, bubbled with 5% CO_2_ and 95% O_2_.

Brain slices were transferred to a recording chamber attached to a microscope (Olympus BX51WI Microscope, Olympus Plan N 4×/0.10 Objective, Olympus LUMPlanFL N 60×/1.00 W Objective, Olympus TH4-200 infrared lamp, Olympus Aplanat Achromat 1.4 NA Oil Condenser and LEJ LQ-HXP-120-3 fluorescence lamp) and continuously perfused with extracellular solution containing 50 μM D-AP5 [D-(-)-2-amino-5-phosphonopentanoic acid] and 20 μM DNQX (6,7-dinitroquinoxaline-2,3-dione; Biotrend) to block glutamatergic transmission. Voltage-clamp recordings were carried out at near-physiological temperature (33–36°C) and current-clamp recordings at room temperature. Cells were visualized and imaged with an Orca R2 CCD camera (Hamamatsu). Voltage- and current-clamp recordings were performed with an EPC9 and EPC10 triple-patch amplifier (HEKA Elektronik). For voltage-clamp recordings access resistance was compensated to residual of 3–4 MΩ of the initial value; data was acquired at a sample rate of 20 kHz and filtered at 2.9 kHz. For whole cell voltage-clamp recordings an internal solution (pH 7.2) consisting of 105 mM Cs gluconate, 26.7 mM CsCl, 10 mM HEPES, 20 mM TEA-Cl, 5 mM Cs-EGTA, 3.3 mM MgCl_2_, 2 mM Na_2_-ATP, 0.3 mM Na_2_-GTP, and 3 mM Na_2_-phosphocreatine was used. For whole cell current-clamp recordings the internal recording solution (pH 7.2) consisted of 145 mM *K*-gluconate, 5 mM KCl, 15 mM HEPES, 2 mM Mg-ATP, 2 mM K-ATP, 0.3 mM Na_2_-GTP, 7.5 mM Na_2_-phosphocreatine, and 5 mM K-EGTA. Transduced neurons were visually identified by their fluorescence (CatCH-mCherry or NpHR3.0-EYFP) and accessed with glass pipettes (resistance between 3 and 5 MΩ). Light stimulation was delivered either by a KSL 083 (590 nm) LED or a KSL 010 (470 nm) LED controlled by a KSL Duo LED controller (Rapp OptoElectronic).

### Analysis of *in Vitro* Data

Electrophysiological data were analyzed with IGOR Pro (WaveMetrics) with the Patcher’s Power Tools 2.19 extension^2^, Excel 2013 (Microsoft), Graphpad Prism 5 (Graphpad Software) and Sigmaplot 11.0 (Systat Software). Latencies were measured as the time from trigger onset to AP peak; only APs that appeared within 30 ms after trigger onset were considered successful. Vector strength (*r*) was calculated using the following formula (Goldberg and Brown, 1969):

$$r\;=\;\frac{\sqrt{{\left(\sum cos\;\theta_{i}\right)}^{2}\;+\;{\left(\sum sin\;\theta_{i}\right)}^{2}}}{n}$$

The jitter is equal to the standard deviation (*SD*) of AP latencies calculated according to the following formula:

$$Jitter\;=\;sd\;=\;\sqrt{\frac{\sum {\left(x\;-\;\bar{x}\right)}^{2}}{\left(n\;-\;1\right)}}$$

### *In Vivo* Electrophysiology

#### Surgical Procedures

Three to 4 weeks after injection of AAV8(Y733F), gerbils were anesthetized with an initial intraperitoneal injection (0.5 ml/100 g body weight) of a physiological NaCl solution (Ringer’s solution) containing 20% (v/v) ketamine chloride (Ketavet, Zoetis Deutschland GmbH) and 2% (v/v) xylazine chloride (Rompun, Bayer HealthCare AG). Depth of anesthesia was monitored by testing the hind leg withdrawal reflex. During recording, anesthesia was maintained at a rate of 2.4 μl per 100 g body weight per minute by means of an automatic pump (801 Syringe Pump; Univentor). Animal body temperature was monitored and maintained using a thermostatically controlled heating pad (Fine Science Tools). Skin and tissue covering the upper part of the skull were removed carefully and a small metal rod was mounted on the frontal part of the skull using UV-sensitive dental-restorative material (Charisma, Heraeus Kulzer). The tragus on both ears was incised to ease access to the ear canal, and headphones (Etymotic Research ER4-PT) were placed into the ear canals. Thereafter, the gerbil was transferred into a sound-attenuated chamber and the animal’s head was fixed in a custom-made stereotaxic device (Schuller et al., 1986). The animal’s head position in the recording chamber was aligned by stereotactic landmarks on the surface of the skull (Loskota et al., 1974). To enable electrodes to penetrate into the DNLL, a craniotomy and durotomy was performed behind the sinus transversus lateral to midline. During the recording session, Ringer’s solution was applied to the opening to prevent dehydration of the brain. We labeled the last recording site by a current-induced lesion (25 μA, 90 s) using metal electrodes (WPI Stimulus Isolator A360). Finally, the animal was killed by intraperitoneal injection of pentobarbital as described above.

#### Light Stimulation

An optic fiber (Thorlabs Multimode Optical Fiber, 0.39 NA, Ø = 200 μm Core, HighOH) was inserted into a cannula with the upper end glued into a ceramic ferrule (Thorlabs FC/SC Ceramic Ferrule, Ø = 2.5 mm, multimode, 230 μm hole-Ø). The fiber was then connected to the LED light source (Thorlabs Fiber Coupled High Power LED, Blue-470 nm or Orange-617 nm, SMA, 1000 mA) via 2 m of connectorized optic fiber and mating sleeve (Thorlabs Ceramic Split Mating Sleeve for Ø = 2.5 mm Ferrules). LEDs were controlled with a 4-Channel LED Driver (Thorlabs) and Single LED Connector Hub (Thorlabs) and triggered and intensity-adjusted with a patch-clamp EPC10 amplifier (HEKA). The cannula with the fiber was mounted on a micromanipulator (IVM single axis, Scientifica) and placed either within 100–350 μm proximity of the recording site or exactly contralateral to it.

#### Extracellular Single-Cell Recording

We recorded single-unit responses utilizing glass electrodes filled with 1 M NaCl (∼7–10 MΩ). The electrodes were maneuvered by a single-axis micromanipulator (Scientifica). Extracellular APs were recorded with a patch-clamp amplifier (EPC10; HEKA) and converted by a RZ-6 Multi I/O Processor (Tucker-Davis Technologies). Residual line noise was removed with a noise eliminator (Humbug, Quest Scientific). AP isolation (signal-to-noise ratio > 3) was performed online by visual choice (AP amplitude) and offline by spike cluster analysis based on stable peak amplitudes and spike waveforms (Brainware, Jan Schnupp, TDT).

#### Acoustic Stimulation

Frequency responses were calibrated for the headphones. Acoustic stimuli were generated digitally, converted to an analog signal at ∼200 kHz sampling rate (RZ-6 Multi I/O Processor, Tucker-Davis Technologies), attenuated and conveyed to the headphones (ER4-PT, Etymotic Research). The standard search stimulus presented to the contralateral ear was a broadband stimulus (white noise bursts) with a duration of 200 ms and squared-cosine rise/fall times of 5 ms. The same stimulus with a duration of 20 ms was presented on the ipsilateral ear after the first 100 ms. The inter-stimulus interval for each repetition was 900 ms. For all recordings, stimulus presentation was pseudo-randomized. Acoustically evoked responses were searched for by administering binaurally delivered noise stimuli without interaural time and intensity differences. A neuron’s best frequency (BF) and threshold were determined by means of binaurally identical (interaural intensity difference/interaural time difference = 0) sinus tone stimulation. BF was defined as the frequency that elicited responses at the lowest sound intensity. Threshold was assigned to the lowest sound intensity evoking a noticeable response at BF. All stimuli applied in the course of our experiments were based upon these characteristics.

### Statistical Analysis

All data are given as either original measurements or mean/median. Statistical analysis was performed using Prism 7 (GraphPad Software, Inc., La Jolla, CA, United States). The statistical tests applied are mentioned in the Figure legends.

## Results

### AAV8(Y733F).hSyn Enables Neuron-Specific Expression of Transgenes in the Gerbil Brainstem and Midbrain

**FIGURE 1:**
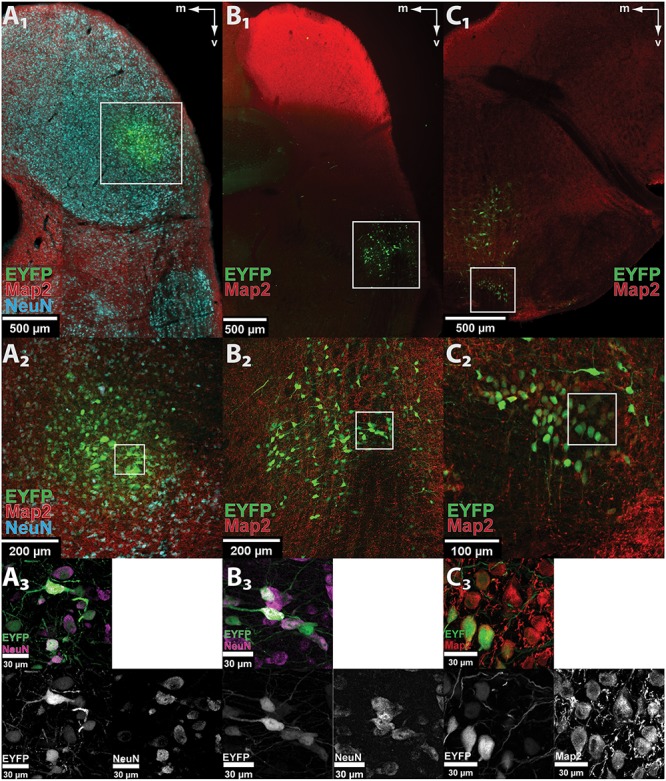
AAV8(Y733F) drives neuron-specific EYFP expression in auditory nuclei. **(A_1_–C_1_)** Low-magnification images of EYFP fluorescence (green) showing correct targeting of the control vector AAV8(Y733F).hSyn.EYFP to the IC **(A_1_)**, DNLL **(B_1_)**, and MNTB **(C_1_)**. The pictures show scans of fixed coronal brain sections taken with the virtualSlide epi-fluorescence microscope. The white frames mark the transduction sites shown enlarged in **(A_2_–C_2_)**, respectively. **(A_2_–C_2_)** Maximum projection of a confocal z-stack taken at 20× optical magnification from the IC **(A_2_)**, DNLL **(B_2_)**, and MNTB **(C_2_)**. The white frames mark the areas shown at higher magnification in **(A_3_–C_3_)**, respectively. **(A_3_–C_3_)** Further magnification (63× objective) reveals neuron-specific expression in IC **(A_3_)**, DNLL **(B_3_)**, and MNTB **(C_3_)**. All EYFP-expressing cells are co-labeled with neuronal markers (Map2, red or NeuN, cyan) as seen in the overlay (top left) and the indicated single channels. Note that not all neurons in the field of view are transduced. m, medial; v, ventral.

The capacity of AAV8(Y733F) to stably and specifically transduce neurons in the gerbil was tested by stereotactically injecting 250 nl of the control vector AAV8(Y733F).hSyn.EYFP, which expresses EYFP under the control of the hSyn promoter, into three different nuclei of the auditory system (IC, DNLL, and MNTB) in anesthetized animals (P 28–60) (Keplinger, 2016). After the indicated expression periods, animals were sacrificed by administering a lethal dose of pentobarbital (IC: 7, 14, 21, and 28 dpi; DNLL: 28 dpi; MNTB: 21 dpi). In all three nuclei, expression of the control fluorophore EYFP was found in coronal sections (50 μm thick) of fixed brains (**Figure 1**). A 100% success rate was achieved in the IC (*n* = 12) and about one-third of injections in the DNLL and MNTB were correctly targeted. The area of transduction was always detected focally around the injection site (IC, *n* = 12/12; MNTB, *n* = 3/3; DNLL, *n* = 3/3) and no evidence for anterograde or retrograde transport was observed. Additional expression of EYFP was detected in the ECIC (*n* = 3/12) upon injection into the lateral part of the central IC (CIC), and along the injection tract when virus particles were injected into the MNTB (*n* = 1/3) or into the DNLL (*n* = 1/3) (data not shown). All transduced, EYFP-expressing cells showed co-labeling with the neuronal markers Map2 and/or NeuN. As a negative control, a glial scar caused by bolus injection of 250 nl of AAV8(Y733F).hSyn.EYFP into the IC displayed extensive labeling of the S100B marker specific for astrocytes at 14 dpi, but no EYFP expression was detected in these cells (Supplementary Figure S1).

### Efficiency of AAV8(Y733F).hSyn.EYFP Transduction and Expression

**FIGURE 2:**
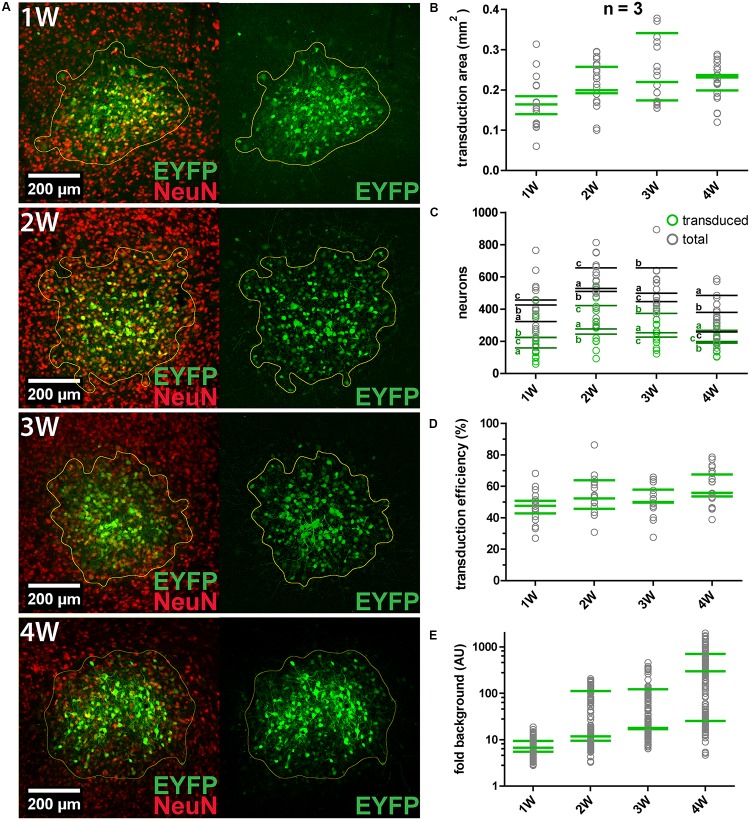
AAV8(Y733F) transduction efficiency and changes with expression duration. **(A)** Representative images obtained at 1, 2, 3, and 4 weeks (1, 2, 3, and 4 W, respectively) after AAV8(Y733F) injection into the IC. Images on the left show the overlays of maximum projections of EYFP (green) and NeuN (red). On the right, sum projections from confocal stacks in the corresponding EYFP channel showing the calculated transduction areas (outlined in yellow) are shown. **(B)** Mean transduction area per animal (*n* = 3) (green strokes). Gray circles depict values for individual slices (*n* = 5-7 slices). **(C)** Number of transduced (green squares) and total neurons (gray squares) for all replicates. Green and gray horizontal strokes mark the mean number of transduced or total neurons, respectively. Individual animals for each time point are labeled with a, b, and c. **(D)** Transduction efficiency (%), i.e., the percentage of transduced neurons inside the transduction area for each replicate (gray circles) and averaged per animal (green strokes). **(E)** The signal-to-noise ratio (SNR) plotted as “fold background” increases significantly from 1 to 4 weeks (Kruskal–Wallis test, *P* = 0.0108; Dunn’s multiple comparison test, *P* = 0.0279). Note the logarithmic scaling of the *y*-axes. Green horizontal strokes mark the mean per animal, gray circles represent individual replicates.

We constantly observed that not all neurons within the transduction area were transduced. For instance, about half of MNTB neurons within the target area were transduced by a single virus injection of 250 nl (*n* = 2). Thus, the transduction efficiency was evaluated by quantification of the number/fraction of transduced neurons, the size of the transduction area and the signal-to-noise ratio (SNR) (**Figure 2**). AAV8(Y733F).hSyn.EYFP was injected into the IC of three animals (P28–P49) for each expression period tested (1, 2, 3, and 4 weeks). At least five coronal sections (50 μm thickness) per animal were immunohistochemically labeled NeuN (**Figure 2A**). The IC was chosen since we observed a positive expression result in 100% of injections. EYFP expression was already detectable 1 week after injection. We counted transduced and total neurons in the transduction area by analyzing the sum projection of the confocal z-stacks (**Figure 2C**). Including all time points, we manually counted 17579 transduced neurons in a total sample of 32369. At all four time points, roughly half of the neurons within the injection area (average over all time points, 54%) were transduced (**Figure 2D**). The size of the transduction area was determined by thresholding the mean intensity for each section (**Figure 2B**). None of these parameters changed significantly with the expression time.

To quantify expression levels in transduced cells over time, the SNR was measured and expressed relative to the background fluorescence (“fold background”; **Figure 2E**). For each biological replicate the average EYFP intensity of 52 cells was measured in the sum projection of a confocal z-stack. The SNR was found to increase over time, with the highest values and highest dynamics being recorded at 4 weeks after injection. In summary, longer expression times resulted in significantly increased expression levels in individual cells (at 4 weeks 48 times higher than at 1 week after injection), but not in significantly greater numbers or fractions of transduced neurons.

### Optical Modification of Neuronal Activity in Acute Gerbil Brain Slices

We performed whole-cell patch-clamp recordings in both current-clamp and voltage-clamp modes to assess the ability of the channelrhodopsin/halorhodopsin to excite/inhibit transduced neurons in response to light. By measuring the photostimulated responses in acute brain slices, we confirmed the functional expression of the transgenes in the plasma membrane. We then characterized the properties of the optogenetic tools as a function of the biophysical properties of the neurons transduced, by determining the levels of functional transgene expression and the effectiveness of the optical stimulation. Animals (P2–P30) were injected with AAV8(Y733F) vectors and sacrificed after 10–28 dpi for preparation of coronal sections of 150–200 μm thickness. Glutamatergic transmission in the slices was blocked by means of D-AP5 and DNQX.

#### Light-Induced Generation of APs in IC and MNTB Neurons Expressing CatCH

**FIGURE 3:**
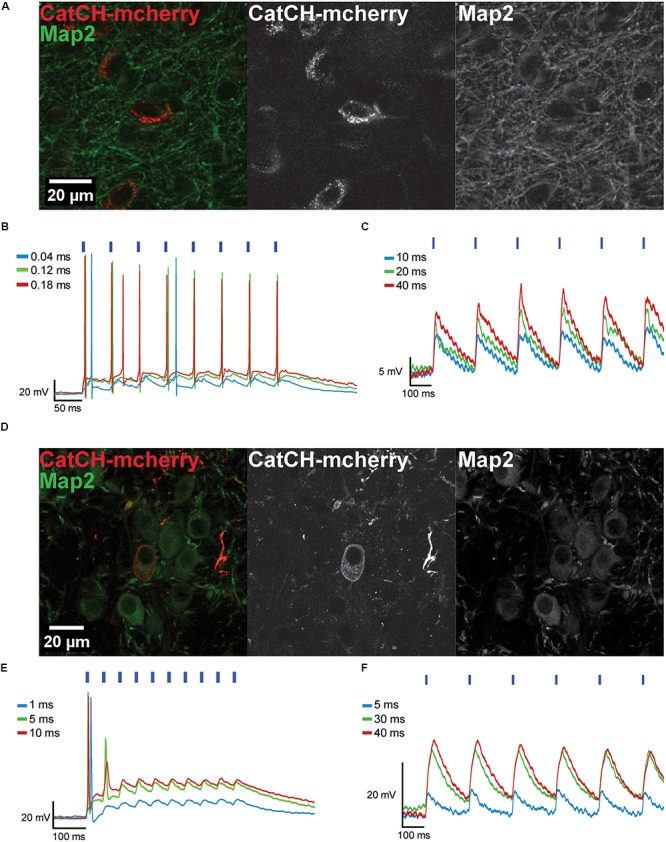
Transduced IC and MNTB neurons expressing CatCH-mCherry can be depolarized with blue light. **(A)** The maximum projections from a confocal z-stack show transduced IC neurons expressing CatCH-mCherry (red). Green fluorescence reveals the Map2 marker. **(B)** Transduced IC neurons fire when stimulated with light pulses of varying pulse widths (40 μs, blue trace; 120 μs, green trace, 180 μs, red trace) at 20 Hz. **(C)** Voltage traces of an IC neuron photostimulated at 5 Hz with pulses of varying durations (10 ms, blue trace; 20 ms, green trace; 40 ms, red trace) show only sub-threshold responses. **(D)** Maximum projections from a confocal z-stack depict transduced MNTB neurons expressing CatCH-mCherry (red). Green labeling represents immunostaining of Map2. **(E)** Transduced MNTB neurons fire when stimulated with light pulses of varying pulse widths (1 ms, blue trace; 5 ms, green trace; 10 ms, red trace) at 20 Hz. **(F)** Voltage traces of a MNTB neuron stimulated at 5 Hz with light pulses of different widths (5 ms, blue trace; 30 ms, green trace; 40 ms, red trace) show only sub-threshold responses. **(B,C,E,F)** Blue strokes above voltage traces indicate the number and onset timing of the light pulses. Width of blue strokes is not to scale but enlarged for better visibility.

The AAV8(Y733F).hSyn.CatCH.mCherry vector was injected into the IC (**Figure 3A**) and into the MNTB (**Figure 3D**), and transduced neurons were identified in brain slices based on their mCherry fluorescence (Supplementary Figure S2). The fluorescence was distributed in a spotty pattern pointing at expression in the plasma membrane (**Figures 3A,D**). Accordingly, 470 nm light pulses elicited APs in 11 out of 21 recorded cells after 10 dpi (**Figures 3B,E**). The remaining neurons were responsive to light, but even after 28 dpi only sub-threshold depolarizations were recorded (**Figures 3C,F**). As APs could be evoked by current injections in these latter neurons (data not shown), we assume that expression levels of CatCH were too low for optical activation.

As crucial prerequisite for *in vivo* utilization of CatCH in neuron populations of the gerbil brain, we examined the dependency of CatCH responses on the properties of the light pulse (optical stimulation frequency, 5–100 Hz; light-pulse widths, 40 μs – 95 ms) and neuronal biophysics (input resistance). Optical excitation was considered to be effective when each light pulse elicited one AP. Any additional evoked spike was counted as an extra spike. The temporal precision of the cellular responses upon effective light stimulation was also quantified. Frequently, not all stimulation protocols and combinations of light-pulse widths and frequencies could be applied to every cell, as some did not survive for long enough.

**FIGURE 4:**
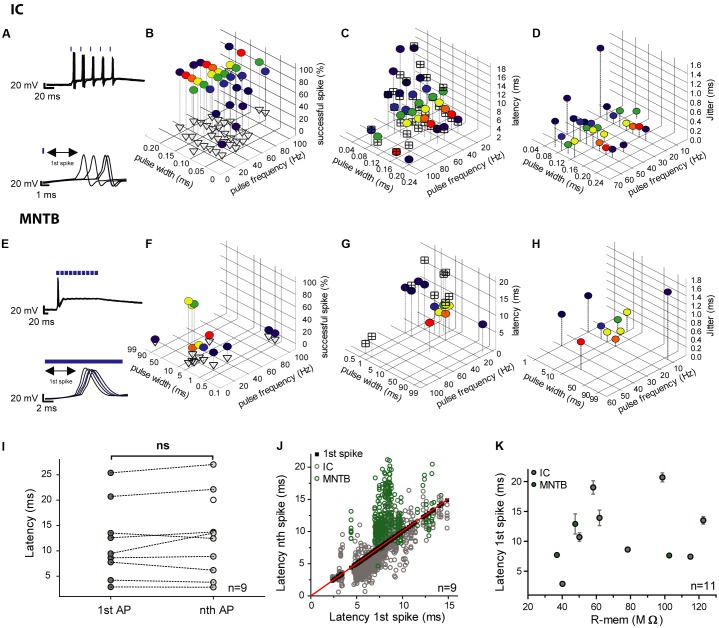
Efficiency, latencies and jitter of light-elicited APs in transduced IC and MNTB neurons. **(A,E)** In each of the the upper panels an overlay of voltage traces for two photostimulated neurons (5 trains at 50 Hz) is depicted. The lower panels show the respective 1st spikes of each of the pulse trains (IC, **A**; MNTB, **E**). The blue strokes above the traces indicate the light pulses. **(B,F)** The graphs show the reliability of photostimulation (in %) for different pulse widths (IC cell 1: 0.04–0.2 ms; IC cell 2: 0.1–20 ms; MNTB cell: 0.5–95 ms) and stimulation frequencies (5–100 Hz). The percentage of successfully elicited spikes within pulse trains is represented by colored circles and that of excess spikes by empty triangles. **(C,G)** Latencies of the first elicited spike (colored circles) and average latencies of all succeeding spikes within pulse trains (crossed squares) over a range of pulse widths (IC cell 1: 0.04–0.2 ms; IC cell 2: 0.1–20 ms; MNTB cell: 0.5–95 ms) and frequencies (5–100 Hz). **(D,H)** Jitter of the first spike (colored circles) over a range of pulse widths (IC cell 1: 0.04–0.2 ms; IC cell 2: 0.1–20 ms; MNTB cell: 0.5–95 ms) and frequencies (5–100 Hz). **(I–K)** Analysis of the correlation between the first and subsequent light-stimulated spikes across all conditions for each individual cell (6 IC neurons; 3 MNTB neurons). **(I)** The mean latencies of the first spike (1st AP; 9 individual neurons: 6 IC, 3 MNTB) does not differ from those of subsequent spikes (nth AP; *p* = 0.39; paired two-tailed *t*-test). **(J)** Latencies of all subsequent light-evoked spikes (nth) plotted against the respective first spike (1st; 6 IC neurons, gray; 3 MNTB neurons, green). Black squares (along the red bisecting line) represent the 1st spike, whereas empty circles represent succeeding spikes. **(K)** The latencies of the first spike averaged across all tested conditions do not correlate with the cells’ input resistance (R-mem; 8 IC neurons, gray; 3 MNTB neurons, green; Spearman’s correlation test, *p* = 0.6538, *r* = 0.1545). The error bars correspond to the standard deviation of the first spikes’ latencies, which is equal to the jitter.

We analyzed the first spike of the train because of the absence of any spike history from preceding spikes (**Figures 4A,E**). Longer light pulses were more efficient, but also evoked extra spikes with a higher probability (**Figures 4B,F**). The shortest achievable spike latencies varied between individual cells. Usually, latencies became shorter with increasing light pulse duration and vice versa (**Figures 4C,G**). To quantify temporal fidelity, the level of jitter of photo-stimulated spikes was determined, and found to be in the (sub-) millisecond range (0.14–9.8 ms). In general, it decreased with increasing pulse duration (**Figures 4D,H**). The latency of the first spike did not differ significantly from those of the succeeding spikes and its mean value was 12.9 ms ± 6.6 ms (*SD*; *n* = 9) for the first one and 14.0 ms ± 7.2 ms (*SD*; *n* = 10) for the subsequent spikes (**Figures 4I,J**). The shortest latency of the first spikes measured for all conditions was 2.3 ms (20-ms light pulse at 5 Hz) and the longest was 32.7 ms (5-ms light pulse at 10 Hz). The latency was independent of the membrane resistance (**Figure 4K**). We calculated the vector strength *r* as another measure of temporal precision for a range of frequencies and pulse widths, in order to identify possible resonance effects that would allow more precise spiking at certain stimulation frequencies. Assuming that resonance is present, stimulation at the resonance frequency would be expected to be more efficient. However, no facilitation by resonance was observed and light-pulse duration turned out to be the dominant factor determining temporal precision (data not shown).

#### Light-Induced Inhibitory Currents and Suppression of APs in IC and MNTB Neurons Expressing NpHR3.0

**FIGURE 5:**
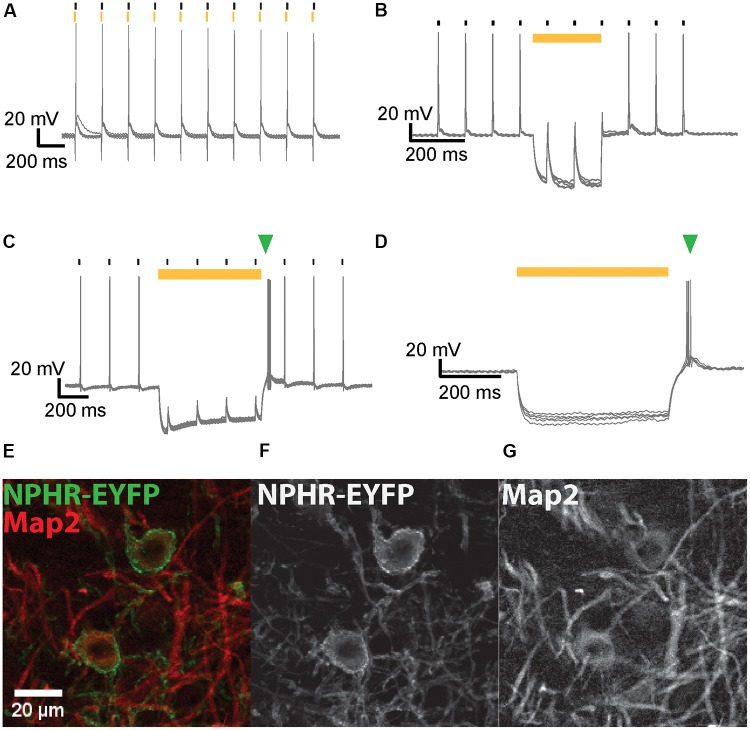
NpHR3.0-mediated suppression of AP generation in IC and MNTB neurons. **(A)** Overlay of voltage traces for an IC neuron expressing NpHR3.0-EYFP when stimulated with current injections (black strokes) immediately after 5-ms light pulses (yellow strokes) applied at 10 Hz. The neuron generates an AP upon current injection despite its light-evoked hyperpolarization. **(B)** A 200-ms light pulse (yellow bar) effectively suppresses spiking of IC neurons in response to current injections (black strokes) at 10 Hz. **(C)** AP generation induced by current injection (black strokes) in a transduced MNTB neuron is suppressed by a 250 ms light pulse (yellow bar). An extra spike (green triangle) is evoked after termination of the light pulse. **(D)** A 500-ms light pulse (yellow bar) induces hyperpolarization of an IC neuron expressing NpHR3.0-EYFP, but the neuron promptly spikes at the end of the light pulse (green triangle). **(E)** Exemplary DNLL neurons exhibit co-localization of NpHR3.0-EYFP (green) and Map2 (red). The image represents the sum projection of a confocal z-stack imaged with the 63× objective. The individual channels for NpHR3.0-EYFP **(F)** and Map2 **(G)** are shown as well.

**FIGURE 6:**
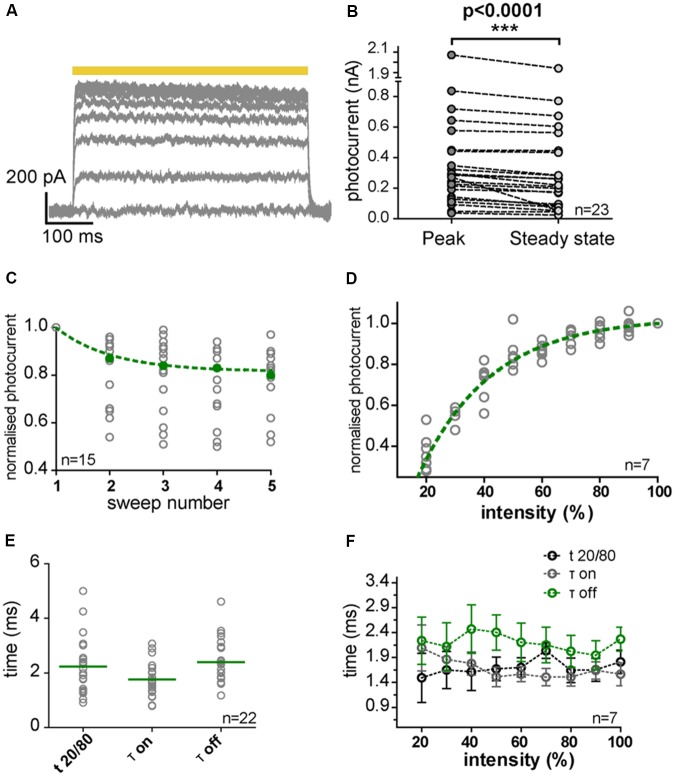
NpHR3.0 photocurrents in transduced IC and MNTB neurons. **(A)** Photocurrent traces evoked in a transduced IC neuron by 500-ms light pulses (yellow bar) with increasing light power (10% steps). The photocurrents saturated for light powers above 50% of the maximum, and slowly declined during the pulse. **(B)** The decline (over the duration of the pulse) in photocurrents evoked by maximal light power (peak, gray circles; steady-state, light gray circles) in IC (*n* = 15) and MNTB (*n* = 8) neurons was statistically significant (*p* < 0.0001, paired one-tailed *t*-test). **(C)** Photocurrents normalized to the current of the first light pulse (gray circles) decreased with repeated stimulation, and this decline can be described by an exponential decay (green dashed line). The green filled circles represent the median of the normalized photocurrents. **(D)** Photocurrents normalized to the response at maximal light power (gray circles) depend on light intensity (Spearman’s correlation test, *r* = 1, *p* < 0.0001) in an exponential fashion (green dashed line). **(E)** Kinetics of the photocurrents as characterized by *t*_20/80_, τ_on_ and τ_off_ measured at maximal light power. Gray circles represent individual measurements and green horizontal strokes depict the mean values. **(F)** Photocurrent kinetics was independent of light intensity (Spearman’s correlation test: *t*_20/80_, *r* = 0.6000; *p* = 0.0968; τ_on_, *r* = –0.6333; *p* = 0.0760; τ_off_, *r* = –0.3333; *p* = 0.3853). Error bars indicate the standard deviation.

In order to hyperpolarize transduced cells with light and thereby suppress APs, AAV8(Y733F).hSyn.NpHR.EYFP was injected into the IC and MNTB. Experimental conditions were similar to those described above. Transduced neurons were identified for electrophysiological recordings by their EYFP fluorescence. NpHR3.0 was activated with orange light pulses (617 nm) of 500 ms duration. The membrane potential was recorded in the current-clamp mode (**Figure 5**) and photocurrents were examined in the voltage-clamp mode (**Figure 6**).

Neurons were stimulated with a train (10 Hz) of current injections each 10% above AP threshold, and light pulses of varying duration were simultaneously presented to suppress spikes (**Figure 5**). At 10 dpi NpHR expression levels were already sufficiently high to inhibit spiking. When the light pulse did not overlap the current stimulation, suppression was ineffective (**Figure 5A**). Photosuppression of individual spikes was successful in only one out of the five neurons tested, and less efficient than the suppression of spike trains by longer light pulses. In most neurons, efficient photosuppression was achieved with light pulses whose onset preceded the current injection by several ms and outlasted it by the same margin (**Figure 5B**). Post-inhibitory rebound spikes due to light-induced hyperpolarization were frequently observed (**Figures 5C,D**).

The elicited photocurrent exhibited a strong dependency on light intensity and increasing the light power resulted in its saturation (**Figures 6A,D**). A decrease in efficiency was observed during stimulation (**Figures 6A,B**), due to depletion of 11-*cis*-retinal by photoconversion. The maximum elicited photocurrent in IC (*n* = 15) and MNTB neurons (*n* = 8) varied widely from cell to cell (**Figure 6B**). The mean peak photocurrent attained at the maximum light power used (6.2 mW/mm^2^; **Figure 6B**) was 390 pA ± 424 pA (*SD*) and the mean steady-state value was 344 pA ± 408 pA (*SD*). A slight decrease in photocurrent was observed for successive sweeps, but this quickly leveled off (**Figure 6C**).

The kinetics of photocurrent induction were analyzed by calculating time constants for ON (tau_on_) and OFF reactions (tau_off_) based on exponential fits (**Figures 6E,F**). The rise time (*t*_20/80_) for an increase in the peak photocurrent from 20 to 80% of its maximum was determined as well, since noise perturbations at 50 Hz had occurred in some of the recordings, and *t*_20/80_ was less affected by this noise. For the maximal light power, the mean values obtained were: tau_on_ = 1.7 ms ± 0.6 ms (*SD*), *t*_20/80_ = 2.2 ms ± 1 ms (*SD*), and tau_off_ = 2.4 ms ± 0.8 ms (*SD*) (**Figure 6E**). These kinetic parameters were independent of the intensity of the light pulse (**Figure 6F**).

### NpHR3.0-Mediated Photosuppression Efficiently Modifies Acoustically Evoked Responses in the IC and the DNLL of Anesthetized Gerbils

We then recorded extracellular potentials of NpHR3.0-expressing neurons in the IC and the DNLL of anesthetized gerbils while simultaneously presenting broadband noise stimuli and pulsed light. Animals were stereotactically injected on P28 or later with AAV8(Y733F).hSyn.CatCH.mCherry.T2A.NpHR3.0 or AAV8(Y733F).hSyn.NpHR3.0.EYFP. The DNLL was chosen as the primary experimental target for *in vivo* optogenetic experiments in order to elucidate its role in the phenomenon of precedence, as outlined in the section “Introduction.” The IC was targeted because of its physical size, its accessibility (short insertion distances for both virus injection and optical fiber) and because the firing patterns of its neurons upon auditory stimulation have been well characterized. These experiments were based on parameters (e.g., light-pulse width and timing) determined for effective optical silencing with NpHR 3.0 in our patch-clamp experiments on acute brain slices. We first set up light delivery into the transduced area and then collected *in vivo* evidence for the impact of light on the firing behavior of stimulus-responsive neurons. Technically, injection of the rAAV vectors, insertion of the optical fiber and positioning of the recording electrode are more demanding for the DNLL than for the IC. Expression times of at least 4 weeks were chosen in order to obtain high expression levels of NpHR3.0. We maximized the volume containing transduced neurons by administering three consecutive virus injections (accepting a slightly increased probability of off-target transduction). The fluorescence emitted by the expressed NpHR3.0-EYFP was concentrated at the plasma membrane and detectable in axons and dendrites of transduced neurons (**Figures 5E–G**).

**FIGURE 7:**
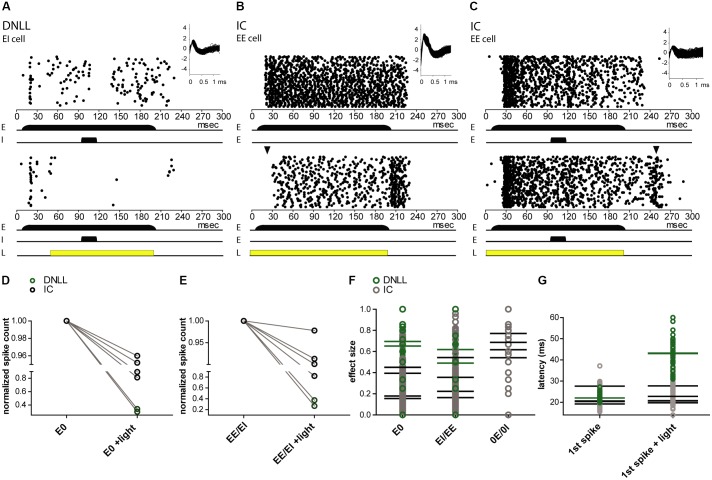
Extracellular *in vivo* recordings reveal NpHR3.0-mediated photosuppression of sound-evoked activity in DNLL and IC neurons. **(A–C)** Peristimulus time histogram (PSTH) for extracellular recordings reveal photosuppression of sound-elicited neuronal activity in transduced DNLL **(A,B)** and IC neurons **(C)**. The upper panels depict responses to auditory stimuli: for all neurons shown, a 200-ms noise stimulus (long black bar) was presented to the excitatory ear (E; in the case of the IC neuron the contralateral ear). In addition, in **(A,C)** a 20-ms noise stimulus (shorter black bar) was presented to either the inhibitory ear (I) in the DNLL neuron or to the ipsilateral ear (E) in the case of the IC. Insets in the upper right corner show overlaid spike shapes. The lower panels show the PSTH with an additional light pulse (yellow bar; **A**, 150 ms; **B**,**C**, 200 ms) triggered at 50 ms. **(A)** Note the persistent inhibition in the control condition (top) during and after noise presentation to the inhibitory ear. **(B)** Note that the onset of the response to the auditory stimulus is delayed in the light condition. The black triangle indicates the occurrence of the first spikes in the control condition. **(D–G)** Analysis of photo-suppression of sound-induced neuronal activity. **(D,E)** The number of spikes normalized to the control condition decreased during the light pulse for both monaural (E0; **D**; *p* = 0.0341, paired one-sample *t*-test) and binaural auditory stimulation (EE/EI; **E**; *p* = 0.0337, paired one-sample *t*-test). Black circles represent IC neurons and green ones DNLL neurons. The *y*-axes are split for better visibility of the data points. **(F)** Effect size for the whole duration of evoked auditory responses. **(G)** Latencies of the first (1st spike) in the absence and presence of photo-stimulation. Mean values for individual neurons are represented as horizontal strokes and individual recordings are depicted as circles (black, IC neurons; green, DNLL neurons).

The sensory evoked spiking activity of transduced cells in the DNLL (*n* = 2) and IC (*n* = 4) could be suppressed by light (**Figure 7**). To quantify the level of photosuppression, spike counts during light application were normalized to the control condition. In one DNLL neuron, spike counts were reduced by 98% while auditory stimulation (mono- as well as binaural) was ongoing, and actually preceded the light pulse by 40 ms (**Figure 7A**). In a recording from an EE neuron in the DNLL, a light pulse that preceded the (mono- as well as binaural) auditory stimulation by 9 ms suppressed spiking activity by 63% (**Figure 7B**). After the light pulse, spiking activity increased to control spike rates. The extent of photosuppression varied from cell to cell (1–98%; **Figures 7D,E**), since distances from the tip of the optical fiber and expression levels of NpHR3.0 itself also varied. The two DNLL neurons (**Figures 7A,B**) were suppressed more effectively than the recorded IC neurons (**Figure 7C**). The effect size represents the difference in spike numbers between control and light conditions (normalized to the control condition) during the entire auditory stimulation period (see “Materials and Methods” section). The mean effect size was higher for monaural auditory stimulations (E0, 0.42; 0E/0I, 0.65), whereas for binaural stimulation the mean effect size was 0.38 (EE/EI) (**Figure 7F**). Owing to the method used to calculate effect sizes, these were smaller for light pulses shorter than the auditory stimulation period. The latencies of the first auditory evoked spike were affected in only one of the recordings (**Figure 7G**).

## Discussion

### AAV8(Y733F) Is a Versatile Tool for Neuron-Specific Transduction in the Gerbil

Using AAV8(Y733F) vectors bearing the relatively short hSyn I promoter, we were able to drive neuron-specific transgene expression in the MNTB, DNLL, and IC of Mongolian gerbils, an important auditory model that has hitherto been inaccessible to optogenetic manipulations. More than 50% of the neurons within the targeted region were found to have been transduced, which is comparable to values reported for the mouse brain using AAV2/8 (Cearley and Wolfe, 2006; McFarland et al., 2009; Cardin et al., 2010). Adequate expression levels were detected as early as 7 days after injection, in agreement with expression times of one to several weeks reported in the literature (Cardin et al., 2010; Adamantidis et al., 2011; Yizhar et al., 2011b). In our study, expression persisted for several weeks, becoming slightly more efficient over that period. The success of stereotactic injection, as indicated by expression of the transduced vector, varies with the anatomical location of the nucleus. The lower values for DNLL and MNTB in comparison to the 100% success rate in the IC are most probably attributable to the smaller physical size and deeper positions of the former (∼5.5 mm from lambda for DNLL; 8.2–8.4 mm for MNTB). Moreover, inter-individual differences in cranial anatomy become more important with longer injection paths, further reducing stereotactical precision and efficiency. Their small particle size of only 20 nm facilitates diffusion of AAVs, allowing them to transduce large target areas; even in case of imprecise injections. One advantage of AAV8(Y733F) is its lack of axonal transport [which has been reported for other AAVs in the mouse (Taymans et al., 2007; Zheng et al., 2009; Masamizu et al., 2011; Castle et al., 2014)], which would otherwise have limited targeting specificity.

### Optical Control of Neuronal Activity in IC, MNTB, and DNLL of the Gerbil

Our *in vitro* electrophysiological characterization of transduced brain slices demonstrates that rAAV vectors are a useful tool for functional and neuron-specific expression of CatCH and NpHR3.0 in the gerbil. By 10 dpi, APs could be elicited or suppressed with light. The most important stimulation parameter was pulse duration and to some degree it can be utilized to compensate for variations in expression levels. Longer light pulses decreased the latencies and jitter of elicited APs, making firing not only more efficient but also faster. The latencies determined in our *in vitro* recordings were similar to published values (Kleinlogel et al., 2011). Latencies of the first and subsequent APs exhibited no differences, which is an important prerequisite for precise high-frequency photostimulation. No increase in phase locking to preferred stimulation frequencies was encountered in IC neurons (Langner and Schreiner, 1988); instead, pulse duration was the determinant parameter for phase locking. The use of very long light pulses resulted in extra spikes or depolarization block, which is characterized by a plateau potential, decreasing AP amplitudes and incomplete repolarization. The mechanisms underlying the depolarization block are not completely understood, but most likely large cation currents leading to prolonged membrane depolarization are involved (Bianchi et al., 2012). The degree of depolarization block varies between neuronal cell types. It is assumed to be lowest in neurons with high spiking frequencies and low input resistances (Herman et al., 2014).

The temporal fidelity of photo-excitation using CatCH did not match the high frequency and temporal precision seen in auditory neurons, since light pulse widths limit the achievable maximum frequency. Though some neurons could be driven with light at 100 Hz in the absence of any extra spikes, most neurons reacted only to pulse trains at 40–50 Hz; these frequencies are nevertheless higher than those usually reported for CatCH (Kleinlogel et al., 2011). However, using the vector introduced here for neuron-specific expression in the gerbil brain together with faster channelrhodopsins such as Chronos (Klapoetke et al., 2014), it should be possible to bring stimulation frequencies closer to the physiological maxima of auditory neurons.

The recorded NpHR3.0 photocurrents recorded here, and their kinetics, match published data (Gradinaru et al., 2010). Current amplitudes varied substantially from cell to cell and with light intensities, but kinetics were independent of light intensity. The decrease in photocurrent with repeated stimulation was too small to become detrimental and might be counteracted by flashes of blue light or longer dark periods (Zhang et al., 2007).

### Light-Stimulated Suppression of Sensory Evoked *in Vivo* Firing in Gerbils

We have successfully established *in vivo* optogenetics in the gerbil brain. APs evoked by auditory stimuli in DNLL and IC neurons expressing NpHR3.0 were suppressed by light in anesthetized animals. As already stated for *in vitro* experiments, the duration and timing of light pulses were the most important determinants of efficient optogenetic stimulation. Light pulses triggered shortly before the onset of the sensory stimulus and entirely encompassing it were shown to be the most effective. Light pulses with these properties have also been used in other *in vivo* studies (Felix-Ortiz et al., 2015). However, light transmission into brain tissue, which is accompanied by losses in light power, remains problematic. To enhance photosuppression, saturating light levels have to be applied, because NpHR3.0 transports only one charge per photon. This was observed in our *in vitro* recordings at 50% light intensity (3–4 mW/mm^2^), all of which were obtained from cells less than 100 μm away from the fiber tip. Usually the fiber was 250–600 μm distant from the recording electrode tip and its output usually amounted to only 0.5–4.5 mW/mm^2^, resulting in lower light levels at the recording site. This factor, in combination with cell-to-cell variability in NpHR3.0 expression levels, most probably contributes to the variations in suppression efficacy. Another effect that needs to be carefully considered is post-inhibitory rebound spikes observed after cessation of the light pulse both *in vitro* and *in vivo*. In patch-clamp experiments these always occurred in neurons that exhibited the hyperpolarization-activated current (*I*_h_), which can depolarize the neuron after the end of illumination (Halder et al., 2015). Experimental conditions should account for these side effects and limit the occurrence of rebound spikes to a time frame that lies beyond the actual tested paradigm. This is another reason for choosing a light-pulse duration which more than encompasses that of the tested auditory paradigm. Inter-stimulus intervals must also be sufficiently long to counteract changes in the chloride reversal potential that occur at constant NpHR activity (Raimondo et al., 2012). The chloride reversal potential is maintained by the transporter KCC2, which is present in high levels in the auditory brainstem (Friauf et al., 2011). Therefore, chloride equilibrium is assumed to be quickly reached after NpHR3.0 activation in these nuclei.

## Conclusion

We have successfully designed and validated optogenetic tools for use in the brainstem (MNTB and DNLL) and midbrain (IC) of the Mongolian gerbil, one of the leading model organisms for studies of auditory processes. We have demonstrated the importance of detailed *in vitro* characterization of the optogenetic tools, in terms of kinetic and illumination parameters, in the nuclei in which they will subsequently be used *in vivo*. As shown here, the viral vector AAV8(Y733F) with the hSyn I promoter enables reliable delivery and expression of genes with neuronal specificity and thus paves the way for studies of unanswered questions in auditory neurobiology in freely behaving animals. Moreover, apart from its use in optogenetics, the viral vector should allow almost any transgene smaller than 4.7 kb to be expressed in neurons of the Mongolian gerbil and may even permit genome editing with the help of the CRISPR/Cas9 system.

## Author Contributions

SK and BB performed the experiments. SK, BB, and LK analyzed the data. SM supervised AAV production. All authors were involved in study design and writing of the manuscript.

## Conflict of Interest Statement

The authors declare that the research was conducted in the absence of any commercial or financial relationships that could be construed as a potential conflict of interest.

## Footnotes

1 http://fiji.sc/Fiji

2 https://www.wavemetrics.com/users/tools_aux001.htm

## ABBREVIATIONS

AAV: adeno-associated virus
AP: action potential
CatCH: Ca^2+^-permeable channelrhodopsin
ChR2: channelrhodopsin 2
D-AP5: D-(-)-2-amino-5-phosphonopentanoic acid
DNLL: dorsal nucleus of the lateral lemniscus
DNQX: 6,7-dinitroquinoxaline-2,3-dione
hSyn: human synapsin
IC: inferior colliculus
LSO: lateral superior olive
MAP2: microtubule-associated protein 2
MNTB: medial nucleus of the trapezoid body
MSO: medial superior olive
NpHR3.0: *Natronomonas pharaonis* halorhodopsin 3.0
P: post-natal day
PBS: phosphate-buffered saline
SNR: signal to noise ratio
WPRE: woodchuck hepatitis virus posttranscriptional regulatory element.
