# Supplementary material for: Optogenetic Control of Neural Circuits in the Mongolian Gerbil
Source: Front Cell Neurosci. 2018 Apr 24;12:111. doi: 10.3389/fncel.2018.00111 (PMC5928259; doi:10.3389/fncel.2018.00111)
Supplement: Supplementary file 1 [file Image_1.PDF]

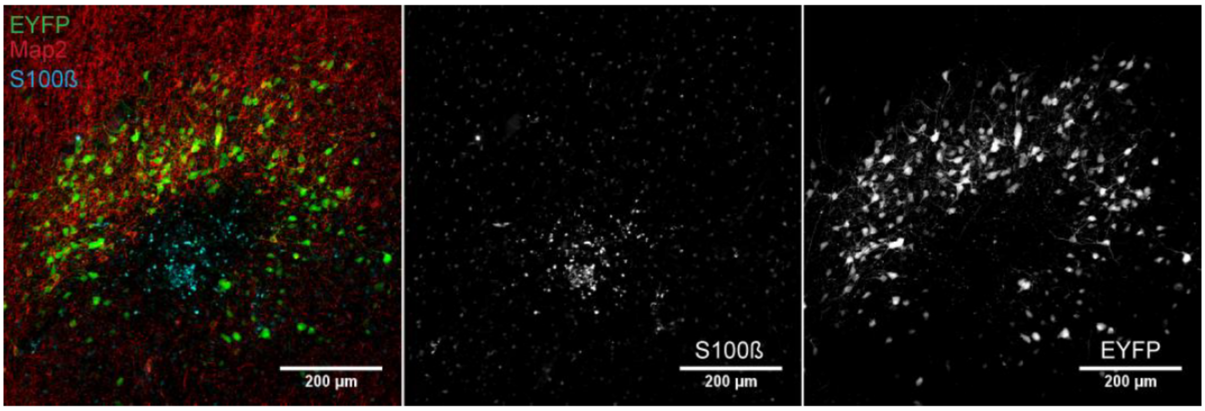

Supplemental Figure S1. rAAV8YF.hSyn.EYFP does not transduce glial cells
Glial cells were abundant at the center of injection 21 dpi after bolus injection of 250 nl *rAAV8YF.hSyn.EYFP*. However, no co-localization of *EYFP* (green) and the glial marker S100ß (cyan) was detected (red, neuronal marker Map2). The left panel shows the overlaid sum projection of confocal z-stacks. The middle and the right panel show the individual channels for S100ß and *EYFP*.

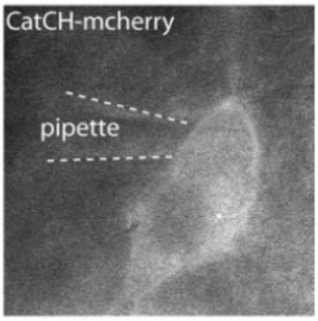

Supplemental Figure S2. Patch pipette accessing an IC neuron displaying mcherry fluorescence
